# A case series of immune checkpoint inhibitor-induced bullous pemphigoid successfully treated with dupilumab and evidence for the BP180 midportion epitope as a preferential autoantigenic target

**DOI:** 10.3389/fmed.2026.1798188

**Published:** 2026-04-01

**Authors:** Dario Didona, Feliciana Mariotti, Anna Pira, Biagio Didona, Michael Hertl, Giovanni Di Zenzo

**Affiliations:** 1 Rare Skin Diseases Center, Istituto Dermopatico dell'Immacolata IDI-IRCCS, Rome, Italy; 2 Laboratory of Molecular and Cell Biology, Istituto Dermopatico dell'Immacolata IDI-IRCCS, Rome, Italy; 3 Universitätsklinikum Marburg, Klinik für Dermatologie und Allergologie, Marburg, Germany

**Keywords:** bullous pemphigoid, cancer, case series, dupilumab, immune checkpoint inhibitor, remission

## Abstract

Bullous pemphigoid (BP) is the most common autoimmune bullous disease and represents a recognized cutaneous immune-related adverse event associated with immune checkpoint inhibitors (ICIs). Management of ICI-induced BP is challenging, as conventional systemic corticosteroids may interfere with antitumor immunity, highlighting the need for effective steroid-sparing therapies. We report a case series of four elderly male patients who developed BP during treatment with ICIs and were successfully managed with dupilumab, an interleukin-4 receptor alpha antagonist. The median age was 72 years, and BP onset occurred a median of 26 weeks after ICI initiation; nivolumab was the trigger drug in all cases. Diagnosis was established through clinical features, histopathology, direct immunofluorescence, and serological detection of IgG antibodies against BP180, including midportion epitopes in some patients. All patients obtained a sustained clinical remission on dupilumab, with a four-year follow-up showing no treatment-related adverse events, no BP relapse despite continued ICI therapy, and no cancer progression. Our findings support the use of dupilumab as a safe and effective steroid-sparing treatment for ICI-induced BP and suggest that non-NC16A BP180 epitopes may aid diagnosis in selected cases.

## Introduction

Bullous pemphigoid (BP), the most frequent autoimmune bullous disease, affects elderly patients and is usually characterized by tense blisters on erythematous skin and extreme pruritus (1). Immune checkpoint inhibitors (ICIs) are monoclonal antibodies targeting the cytotoxic T-lymphocyte antigen-4 (CTLA-4) or programmed cell death-1/ligand 1 (PD-1/PD-L1), which are involved in the negative regulation of T-cell immune function (2, 3). Approximately 30% of patients treated with anti PD-L1 and 50% of those treated with anti CTLA-4 ICIs develop cutaneous immune-related adverse events (irAEs), including BP, which has been extensively reported in the literature following ICI therapy (2, 3). Conventional therapies for BP include high-potency topical corticosteroids, oral prednisone, and steroid-sparing immunosuppressive drugs (1). However, in refractory BP patients or in case of contraindications to conventional therapies, biologic drugs may also be considered as an alternative (1). Dupilumab, recently approved by the United States Food and Drug Administration (FDA) for the treatment of BP, is a fully human IgG4 monoclonal antibody targeting IL-4 receptor alpha that inhibits the signaling of IL-4 and IL-13 (1). Our case series highlights the role of dupilumab as an effective and safe therapy for BP induced by ICIs, leading to a sustained clinical remission without adverse events, no BP relapse despite continued ICI therapy, and no cancer progression. In addition, we pointed out the role of the BP180 midportion epitope as a potential preferential autoantigenic target in BP triggered by ICIs.

## Case description

Here, we reported a case series of four male patients on ICIs, who developed BP and have been successfully treated with dupilumab (Tables 1, 2).

**Table 1:** Demographic, clinical, and immunological characteristics of four patients who developed bullous pemphigoid after therapy with immune checkpoint inhibitors for cancer.

Patient	1	2	3	4
Sex	M	M	M	M
Age at diagnosis^*^ (years)	65	83	76	68
Neoplasia	Melanoma	NSCLC	RCC	Melanoma
Stadium	IV (T3a, N2, M1c)	IVa (T4, N3, M1b)	IV (T3a, N2, M0)	IV (T3a, N3, M1a)
ICI	Nivolumab	Nivolumab	Nivolumab	Nivolumab
DIF/Histology	ND/ND	ND/Pos	Pos/Pos	Pos/Pos
BP180-NC16A^a, b^ (^a^U/ml, ^b^UR/ml)	0.7^a^	8.9^a^	98.1^a^	52^b^
BP230^a, b^ (^a^U/ml, ^b^UR/ml)	3.4^a^	0.3^a^	4.0^a^	28^b^
ECD-BP180^c^ (PIV)	11.53	ND	37.25	ND
E-1080^d^ (PIV)	203.9	ND	163.0	ND
E-1331^e^ (PIV)	2.7	ND	4.9	ND

AA, amino acid; DIF, direct immunofluorescence; ICI, immune checkpoint inhibitor; M, male; ND, not done; NSCLC, non-small cell lung cancer; PIV, pemphigoid index value; Pos, positive; RCC, renal cell carcinoma.

PIV represents the standardization of extinction data on the positive and negative internal standards.

^*^Age at the diagnosis of the neoplasia.

^a^MBL commercial ELISA kit; cut-off ≥ 9.0 U/ml.

^b^Euroimmun commercial ELISA kit; cut-off ≥ 20.0 UR/ml.

^c^In-house ELISA based on the ectodomain of BP180 (AA 490-1497), cut-off ≥ 10.02 PIV.

^d^In-house ELISA based on the mid portion of BP180 (AA 1080-1107), cut-off ≥ 14.9 PIV.

^e^In-house ELISA based on the C-terminal region of BP180 (AA 1331-1404), cut-off ≥ 4.5 PIV.

**Table 2:** Summary of clinical events, interventions, and outcomes in four patients treated with dupilumab for nivolumab-induced bullous pemphigoid.

Patient	Start of nivolumab	BP onset	Therapy for BP–Drug (mg/day)	Start of dupilumab	BP and neoplasia outcome (4-year follow-up)
1	Week 0	Week 32	Prednisone (10) Dapsone (50)	Week 96	Week 305: BP on CR on ongoing therapy with dupilumab. No tumor progression on ongoing therapy with nivolumab
2	Week 0	Week 60	Prednisone (25)	Week 116	Week 325: BP on CR on ongoing therapy with dupilumab. No tumor progression on ongoing therapy with nivolumab
3	Week 0	Week 16	Prednisone (25) Doxycyclin (200) Dapsone (100)	Week 52	Week 261: BP on CR on ongoing therapy with dupilumab. No tumor progression on ongoing therapy with nivolumab
4	Week 0	Week 20	Prednisone (25) Dapsone (100)	Week 40	Week 249: BP on CR on ongoing therapy with dupilumab. No tumor progression on ongoing therapy with nivolumab

BP, bullous pemphigoid; CR, complete remission. Weeks are calculated from nivolumab initiation, which represents the baseline.

### Patient 1

A 65-year-old male White patient was affected by malignant melanoma (MM) stadium IV (T3a, N2, M1c), showing lymph node and brain metastases. He was affected by arterial hypertension, diabetes mellitus type II, and diverticulitis. The patient was treated with nivolumab as adjuvant monotherapy after surgical removal of the malignant melanoma according to the technical schedule of this drug. After 32 weeks on nivolumab, the patient developed tense blisters on erythematous skin, which was associated with intense pruritus (Tables 1, 2).

### Patient 2

An 83-year-old male White patient was affected by non-small cell lung cancer (NSCLC) stadium IVa (T4, N3, M1b), showing lymph node and bone metastases. He was affected by arterial hypertension and dyslipidemia, and he had a previous history of Non-ST-Elevation Myocardial Infarction (NSTEMI). The patient was treated with nivolumab as second-line monotherapy for NSCLC after failure of the previous chemotherapy according to the technical schedule of this drug. After 60 weeks on nivolumab, the patient developed tense blisters on erythematous skin, associated with intense pruritus (Tables 1, 2).

### Patient 3

A 76-year-old male White patient was affected by renal cell carcinoma (RCC) stadium IV (T3a, N2, M0), showing lymph node metastases. He was affected by arterial hypertension, dyslipidemia, and hyperuricemia. The patient was treated with nivolumab as adjuvant monotherapy for RCC after surgical therapy according to the technical schedule of this drug. After 16 weeks on nivolumab, the patient developed erythematous urticarial plaques with intense pruritus (Tables 1, 2).

### Patient 4

**Figure 1:**
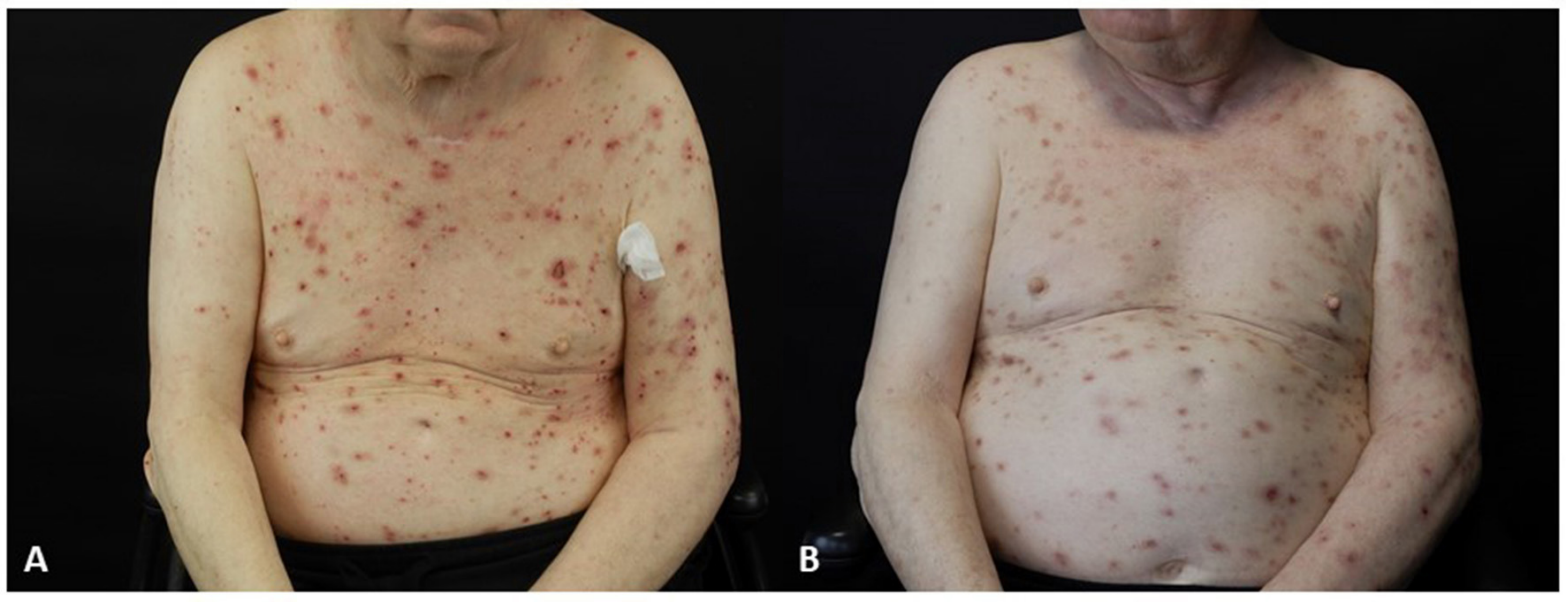
**(a)** Patient 4 before dupilumab administration. **(b)** Patient 4 after dupilumab administration at week 44.

A 68-year-old male White patient was affected by MM stadium IV (T3a, N3, M1a), showing lymph node and bone metastases. He was affected by arterial hypertension and dyslipidemia. The patient was treated with nivolumab as adjuvant monotherapy after surgical removal of the malignant melanoma according to the technical schedule of this drug. After 20 weeks on nivolumab, the patient developed pruriginous nodules on the upper limbs, chest, and abdomen (Figure 1a, Tables 1, 2).

## Diagnostic assessment

### Patient 1

Despite negativity to the immunodominant NC16A epitope, according to the detection of serum IgG targeting the midportion of BP180 (AA 1080-1107) and to the clinical features, the diagnosis of BP was made. The patient was treated with a combination of prednisone 10 mg and dapsone 50 mg because of type 2 diabetes. However, the patient did not respond to the combination therapy and developed more blisters. Therefore, 64 weeks after the development of BP, we decided to continue the administration of nivolumab and to switch the therapy of BP to dupilumab, according to the schedule of atopic dermatitis. The patient showed a massive improvement in clinical picture, and over a 4-year follow-up period, he did not show any progression of MM (Tables 1, 2).

### Patient 2

According to the clinical and histological features, the diagnosis of BP was made. Because of the reduced activity of glucose-6-phosphate dehydrogenase (G6PDH), dapsone was not administered. In addition, the patient had an allergy to tetracyclines, and doxycycline was not considered as therapy. Therefore, the patient was treated with prednisone 25 mg once per day. However, the patient did not respond to the therapy, developed more blisters, and showed a worsening of arterial hypertension. Therefore, 56 weeks after the development of BP, we decided to continue the administration of nivolumab and to switch the therapy of BP to dupilumab, according to the schedule of atopic dermatitis. The patient showed an impressive improvement in clinical picture, and over a 4-year follow-up period, he did not show any progression of NSCLC (Tables 1, 2).

### Patient 3

Histology, direct immunofluorescence (DIF), and detection of IgG against BP180 confirmed the diagnosis of BP. The patient was treated with prednisone 25 mg once per day. However, the patient developed blisters. Therefore, dapsone 100 mg per day was added. Unfortunately, the patient developed exertional dyspnea, and the administration of dapsone was interrupted. Therefore, doxycycline 100 mg twice per day was added as a steroid-sparing agent. However, the clinical picture did not improve. Therefore, 36 weeks after the development of BP, we decided to continue the administration of nivolumab and to switch the therapy of BP to dupilumab, according to the schedule of atopic dermatitis. The patient showed a significant improvement in clinical picture, and over a 4-year follow-up period, he did not show any progression of RCC (Tables 1, 2).

### Patient 4

Histology, DIF, and detection of IgG against BP180 confirmed the diagnosis of BP. The patient was treated with prednisone 25 mg once per day. However, the patient did not improve. Therefore, dapsone 100 mg per day was added. Unfortunately, the patient developed exertional blurred vision, and the administration of dapsone was interrupted. Therefore, doxycycline 100 mg twice per day was added as a steroid-sparing agent. However, the clinical picture did not improve. Therefore, 20 weeks after the development of BP, we decided to continue the administration of nivolumab and to switch the therapy of BP to dupilumab, according to the schedule of atopic dermatitis. The patient showed a significant improvement in clinical picture (Figure 1), and over a 4-year follow-up period, he did not show any progression of MM (Tables 1, 2).

## Discussion

The median age of our cases was 72 years (range: 65–83), and the median time to BP onset after starting ICIs was 26 weeks (range: 16–60). Melanoma was the primary tumor in two of four patients, and nivolumab was the trigger drug in all cases. After a 4-year follow-up, all patients were in clinical remission on ongoing therapy with dupilumab (Figure 1), and none had any adverse events. In addition, after a 4-year follow-up, none of the patients had a progression of the tumor on dupilumab and continuing ICI (Tables 1, 2).

Current therapeutic approaches for ICI-BP are based essentially on the use of systemic corticosteroids and conventional immunosuppressive agents, which can both compromise antitumor immune responses (1). Systemic corticosteroids are also associated with several acute and chronic side effects, and, in addition, a subset of BP on ICIs can show steroid resistance. Consequently, alternative therapeutic strategies are necessary.

Recent studies have demonstrated upregulation of IL-4– and IL-13–associated genes in both spontaneous and ICI-induced BP compared with healthy skin (4), along with marked dermal expression of IL-4 and IL-13 in proximity to the dermo-epidermal junction in lesions of BP on ICIs (5). Furthermore, analyses of samples from patients with BP have shown that treatment with dupilumab results in a reduction of circulating IL-4– and IL-13–producing CD4^+^ Th2 cells (6). These findings provide a strong mechanistic rationale for the use of dupilumab as a therapeutic option in patients with BP triggered by ICIs.

Consistent with this rationale, several studies have reported dupilumab to be a safe and effective treatment for BP induced by ICIs (7–9). Furthermore, a recent study evaluating long-term mortality outcomes in 53 patients with cutaneous irAEs treated with dupilumab reported that overall survival did not significantly differ between dupilumab-treated patients and patients on ICIs without cutaneous irAEs or with cutaneous irAEs who did not receive dupilumab (10). Notably, the use of systemic corticosteroids within 2 years of ICI initiation was associated with poorer overall survival compared with the dupilumab-treated group (10).

In this context, we describe four cases of BP triggered by ICIs successfully managed with dupilumab. Despite the inherent limitations of a case series, such as the small sample and the absence of a control group, a key strength of our case series is the extended long-term follow-up, reaching up to 4 years. During this period, none of the patients experienced tumor progression while receiving dupilumab concomitantly with ongoing ICI therapy. An additional novel observation concerns the potential diagnostic utility of reactivity against the midportion of BP180, which may represent a helpful diagnostic marker, particularly in patients for whom a skin biopsy is unavailable.

Our data align with previous reports. Indeed, several multicenter, retrospective, observational studies in dermatological units have shown that BP following ICI therapy mostly affects male patients with melanoma as the most common primary tumor and nivolumab as the most frequent trigger, followed by pembrolizumab (2, 3). Although the pathogenesis of ICI-induced BP is not completely understood, a putative mechanism can be postulated. ICIs predominantly act on T cells; however, B-cell dysregulation also plays a role in the autoimmune manifestations associated with anti–PD-(L) 1 therapy (11). In fact, ICIs may promote the expansion of autoreactive B cells and the subsequent antibody production (11). As the expression of BP180 is not restricted to the dermo-epidermal junction but has also been identified in certain cancer cells, targeting BP180 on tumor cells may elicit a cross-reactive immune response against the BP180 present in the dermo-epidermal junction, ultimately contributing to BP development (3, 11). Several studies have reported BP autoantibodies recognizing BP180 antigenic sites different from NC16A. A midportion epitope (E-1080: AA 1080-1107) and a C-terminal one (AA 1331-1404) were recognized by almost half of BP patients and in several cases also by NC16A-negative patients (12–14). A recent study aimed at mapping BP180 ectodomain reactivity in NC16A-negative BP patients reported that 98% of sera recognized a region corresponding to the AA 1024–1270 (15). In line with these findings a NC16A-negative patient reacted to E-1080 midportion epitope.

In conclusion, this case series highlights the importance of dupilumab in BP induced by ICI, which may represent a safe therapeutic alternative to conventional treatments, allowing the continuation of ICI therapy in this particularly vulnerable population. The reactivity to the E-1080 suggests that an ELISA based on the mid-portion epitope of BP180 may be useful for diagnosing BP triggered by ICIs in NC16A-negative patients or when a biopsy is unavailable.

## Patient perspective

All patients included in this case series reported a meaningful improvement in their quality of life following dupilumab treatment. Before therapy, the burden of BP significantly impacted their daily activities and overall wellbeing, adding further distress to an already challenging oncological condition.

Following dupilumab initiation, all patients experienced notable relief from pruritus and progressive skin healing, which translated into improved sleep, greater physical comfort, and enhanced ability to carry out everyday activities.

Overall, the patients' perspectives underscore the importance of effective and well-tolerated therapeutic options in this vulnerable population, where quality of life represents a central treatment goal alongside oncological outcomes.

## Data Availability

The raw data supporting the conclusions of this article will be made available by the authors, without undue reservation.
